# Phenotype-dependent apoptosis signalling in mesothelioma cells after selenite exposure

**DOI:** 10.1186/1756-9966-28-92

**Published:** 2009-06-29

**Authors:** Gustav Nilsonne, Eric Olm, Adam Szulkin, Filip Mundt, Agnes Stein, Branka Kocic, Anna-Klara Rundlöf, Aristi P Fernandes, Mikael Björnstedt, Katalin Dobra

**Affiliations:** 1 Karolinska Institutet, Department of Laboratory Medicine, Division of Pathology, Stockholm, Sweden

## Abstract

**Background:**

Selenite is a promising anticancer agent which has been shown to induce apoptosis in malignant mesothelioma cells in a phenotype-dependent manner, where cells of the chemoresistant sarcomatoid phenotype are more sensitive.

**Methods:**

In this paper, we investigate the apoptosis signalling mechanisms in sarcomatoid and epithelioid mesothelioma cells after selenite treatment. Apoptosis was measured with the Annexin-PI assay. The mitochondrial membrane potential, the expression of Bax, Bcl-XL, and the activation of caspase-3 were assayed with flow cytometry and a cytokeratin 18 cleavage assay. Signalling through JNK, p38, p53, and cathepsins B, D, and E was investigated with chemical inhibitors. Furthermore, the expression, nuclear translocation and DNA-binding activity of p53 was investigated using ICC, EMSA and the monitoring of p21 expression as a downstream event. Levels of thioredoxin (Trx) were measured by ELISA.

**Results:**

In both cell lines, 10 μM selenite caused apoptosis and a marked loss of mitochondrial membrane potential. Bax was up-regulated only in the sarcomatoid cell line, while the epithelioid cell line down-regulated Bcl-XL and showed greater caspase-3 activation. Nuclear translocation of p53 was seen in both cell lines, but very little p21 expression was induced. Chemical inhibition of p53 did not protect the cells from apoptosis. p53 lost its DNA binding ability after selenite treatment and was enriched in an inactive form. Levels of thioredoxin decreased after selenite treatment. Chemical inhibition of MAP kinases and cathepsins showed that p38 and cathepsin B had some mediatory effect while JNK had an anti-apoptotic role.

**Conclusion:**

We delineate pathways of apoptosis signalling in response to selenite, showing differences between epithelioid and sarcomatoid mesothelioma cells. These differences may partly explain why sarcomatoid cells are more sensitive to selenite.

## Background

Selenite is a redox-modulating compound which is increasingly investigated for use as an anticancer agent. We have recently shown that selenite induces apoptosis in malignant mesothelioma cells in a dose-, time- and phenotype-dependent manner, with a more potent effect on sarcomatoid cells [1,2]. Promising anti-cancer effects have also been shown in *in vitro *models of lung, prostate, breast, skin, and hematologic cancers [3-12], with a selective effect upon malignant cells compared to normal cells [1,4,13]. Several investigators have showed independently that selenite cytotoxicity can be inhibited by antioxidants [1,14-19]. Redox regulation is likely to influence cellular sensitivity to selenite, and we have reported that selenite decreases the activity of thioredoxin reductase (TrxR) [1]. Together with thioredoxin (Trx) and NADPH, it forms the thioredoxin system, which is highly active in redox signalling and defence against oxidative stress.

Malignant mesothelioma is a tumor of the serosal membranes, most often arising in the pleura after prolonged asbestos exposure. This tumor has a peculiar pattern of differentiation, where the malignant cells may assume either an epithelioid or a sarcomatoid phenotype. These two phenotypes exhibit differences in their biological behavior, as evidenced by gene expression analyses [20-23] and the fact that presence of sarcomatoid cells is associated to poor prognosis and increased therapy resistance [24-26]. The median survival time from diagnosis is around 12 months [27]. Response rates to current pharmacological therapies are low, reaching only 40% at best [28,29].

This study aimed to investigate apoptosis signalling during selenite treatment in an epithelioid and a sarcomatoid mesothelioma cell line. Both were initially derived from the same tumor [30], and the latter is more sensitive to selenite. Thus, we anticipated the emergence of differences in apoptosis signalling in response to selenite that might explain the differential sensitivity of the two cell lines.

## Methods

### Cells and culture

This study was carried out using a well-established model system for mesothelioma differentiation, consisting of the two cell sub-lines STAV-AB and STAV-FCS. Cells were derived from a single tumor, and subsequently induced to differentiate into the epithelioid (STAV-AB) and the sarcomatoid phenotype (STAV-FCS), respectively, by altering the serum composition [30]. Hence, STAV-AB cells were grown in Gibco RPMI 1640 medium (Invitrogen) and 10% human AB serum, whereas STAV-FCS cells were grown in the same medium and 10% fetal calf serum. The specific differentiation of these cells has been evidenced by immunoprofiling showing that STAV-AB cells express more cytokeratin, whereas STAV-FCS cells have stronger reactivity to vimentin antibodies [21] as well as by morphometry. The elongated sarcomatoid cell morphology of the STAV-FCS cells and the more round epithelioid morphology of the STAV-AB cells have been confirmed by average length:width ratios of 3.42 in the STAV-FCS cells and 1.58 in the STAV-AB cells [31]. Jurkat cells were obtained from the American Type Culture Collection (ATCC) and grown in RPMI 1640 medium and 20% fetal calf serum. All cells were grown at 37°C with 5% CO_2 _and passaged approximately twice per week.

### Treatment of cell cultures and inhibition of signalling enzymes

To investigate the contributions of several signalling pathways, inhibitors were used against key enzymes. Cells were washed, trypsinized and re-seeded with the respective inhibitors (specified in table 1) 24 h prior to selenite treatment, except for the JNK-inhibitor, with which they were pre-incubated for 1 h. Selenite was then added to the medium and the cells were harvested 24 or 48 h later. Titrations were performed with all inhibitors based on the manufacturers' instructions and concentrations reported in the literature. In all cases, the highest non-toxic doses tested were accepted.

**Table 1:** Chemical inhibitors against apoptosis signalling enzymes

*Inhibitor*	*Target*	*Concentration*	*Purchased from*
SB 203580	p38	5 μM	Merck
SP 600125	JNK	10 μM	A.G. Scientific
Pifithrin	p53	10 μM	A.G. Scientific
Pepstatin A	Cathepsin D, E	5 μM	Calbiochem
Ca-074 Me	Cathepsin B	10 μM	SERVA Electrophoresis GmbH

### Cell viability assays

Viability assays were performed in conjunction with flow cytometry experiments to obtain internal controls. Aliquots of cell suspensions prepared for flow cytometry were plated in triplicates in 96-well plates, with a density of approx. 5000 cells per well. They were then analysed using the WST-1 assay (Roche), whereby a tetrazolium salt is cleaved by mitochondrial enzymes to yield a coloured product, to measure viability. The plates were read in a Spectramax spectrophotometer at 450 nm with subtraction of background absorbance at 600 nm.

### Flow cytometric analyses

Flow cytometric assays for detection of apoptosis were carried out using the Annexin V kit (Caltag Laboratories) according to the manufacturer's protocol. Briefly, trypsinized cells were resuspended in Binding Buffer with Annexin V-FITC and Propidium Iodide (PI), and incubated for 15 minutes in the dark.

For analysis of Bax expression, cells were fixed in 0.25% paraformaldehyde, and permeabilised with 100 μg/ml Digitonin. Aliquots were then incubated for 30 minutes with phycoerythrin-conjugated mouse anti-human Bax antibody (Santa Cruz, sc20067) or mouse IgG as a control, both in final dilution 1:10. Morphological controls were established using cytospins. Slides were fixed in 4% buffered formaline, washed 2 × 5 min in PBS, and air-dried. Staining was performed with the same antibody concentration and incubation time, and the staining was evaluated by confocal microscopy.

For analysis of Bcl-XL expression, cells were fixed in 1% formaldehyde, and permeabilised with 0.1% Saponin. Aliquots were then incubated for 15 minutes with phycoerythrin-conjugated rabbit anti-human Bcl-XL antibody (GeneTex, GTX46035), final dilution 1:800, or rabbit IgG as a control. The secondary antibody Alexa 488 goat anti-rabbit IgG was diluted 1:1600, and incubation was performed for 15 minutes in the dark.

The mitochondrial membrane potential was measured using two independent methods. 1) The Mitochondria Staining Kit (Sigma) was used according to the manufacturer's instructions. Briefly, cells were trypsinised and then resuspended in a solution of 45% medium, 5% serum and 50% staining solution containing the JC-1 probe. They were incubated for 20 min in 37°C, and then washed with staining buffer. Cells treated with Valinomycin were used as a positive control. 2) With the fluorescent probe DiOC6(3) (3,3-dihexyloxacarbocyanine iodide; Molecular Probes), cells were incubated for 15 minutes with concentrations ranging from 1 to 100 nM DiOC6(3). After staining, an aliquot of cells was prepared for confocal microscopy to verify that the staining was localized to the mitochondria.

For analysis of procaspase-3 expression, cells were fixed in 1% paraformaldehyde, and permeabilised with 10 μg/ml Digitonin. Aliquots were then incubated for 30 minutes with a rabbit monoclonal antibody to procaspase-3 (Abcam, ab32150), final dilution 1:150, or rabbit IgG as a control. The secondary antibody Goat anti-Rabbit IgG-FITC (Abcam, ab6717) was diluted 1:300, and incubation was performed for 30 minutes in the dark.

Detection of the active form of caspase-3 was performed with a FITC-conjugated antibody (BD Biosciences, 559341). Cells were fixed in 1% paraformaldehyde, and resuspended in 100 μg/ml Digitonin solution with antibody in final dilution 3:20, and incubated for 30 minutes at 4°C. Cells treated with 2 μM doxorubicin for 24 h were used as positive controls.

Flow cytometry was always performed immediately after the staining was completed. All analyses were performed on a Becton Dickinson flow cytometer and the data were processed in the Cell Quest program.

### Cytokeratin 18 cleavage assay

The M-30 Apoptosense assay (Peviva, Stockholm) measures cytokeratin 18 fragments specifically produced by cleavage by caspases 3, 6, 7, and 9 [32,33]. Cells were seeded in 96-well microtiter plates with or without 10 μM selenite and 0.2 μg/ml doxorubicin. After 24 h, cells were lysed by the addition of 10 μl 10% Tergitol-type NP-40 (Sigma-Aldrich) to each well. The ELISA analysis was carried out according to the manufacturer's instructions. Briefly, 25 μl samples were incubated together with 75 μl horseradish peroxidase-conjugate solution on the ELISA microplate for 4 h on a shaker. 200 μl of tetramethylbenzidine substrate solution were added and the plate was incubated for a further 20 min. The reaction was stopped by the addition of 50 μl 1.0 M H_2_SO_4_, and the absorbance at 450 nm was determined on a Spectramax spectrophotometer.

### Immunocytochemistry and confocal microscopy

For analysis of nuclear translocation of p53 and p21, cytospins were prepared. For p53 analysis, the slides were fixed in ice-cold dry acetone. Prior to staining, they were heated to 100°C for 5 min in citrate buffer, pH 6.0. Staining was performed using the p53 Refine kit (Novacastra). For p21 analysis, the slides were fixed in 4% buffered formaline, and air-dried. Staining was performed with a mouse monoclonal antibody (Calbiochem, OP64), diluted 1:200, for 30 minutes.

For analysis with monodansyl cadaverine (MDC), cells were grown on sterilised Superfrost Plus slides (Menzel GmbH &Co). The slides were stained for 10 minutes with 10 μM MDC (BioChemica), and immediately analysed by confocal microscopy.

### DNA binding assay for p53

Nuclear extracts were prepared as described previously [34]. Electrophoretic Mobility Shift Assay (EMSA) was conducted using the LightShift Chemiluminescent EMSA Kit (Pierce). 20 μg of nuclear protein was used for each sample. The double-stranded oligonucleotide probes for the p53 binding site (sense 5'-TACAGAACATGTCTAAGCATGCTGGGG-3') were annealed and labeled with biotin. To label DNA probes, the Biotin 3' End DNA Labeling Kit (Pierce) was used according to the manufacturer's protocol.

### Measurement of Thioredoxin

ELISA was used to quantify the amounts of thioredoxin (Trx) in the cells. The assay was adapted from Pekkari et al [35]. Wells were coated with a primary monoclonal antibody (2G11, kindly provided by dr. Anders Rosén of the University of Linköping) overnight at 4°C, 5 μg/ml diluted in carbonate buffer, pH 9.6. Secondary biotinylated antibody (IMCO Corporation) was added in a concentration of 5 μg/ml. Absorbance at 405 nm was measured using a SpectraMax 250 spectrophotometer (Molecular Devices). Data were analyzed using the SOFTmax Pro software, v. 2.6.

### Statistical methods

All experiments were performed at least three times. When one representative experiment is shown, it was chosen on the basis of being closest to the average of all the experiments performed. Student's t-test, two-way ANOVA with Dunnett's post test or Bonferroni's multiple comparison test, and χ^2^-tests were used to determine statistical significance. The choice of statistical method for each experiment is indicated in the respective legend text.

## Results and discussion

Selenite is a strong prooxidant when used in cytotoxic doses, and may induce apoptosis. Many independent researchers have confirmed that the cytotoxicity of selenite is mediated by oxidative stress, in cell types so various as malignant mesothelioma [1], hepatoma [14,15,36], cancers of the breast [16], prostate [4,17,19,37], and uterine cervix [18], glioma [38], lymphoma [39], and Jurkat T-cells [40]. Cell death with apoptotic characteristics has also been observed in erythrocytes following selenite treatment [41]. Selenite-induced oxidation may target many cellular components, and the resulting damage and cell signalling is therefore likely to be dependent on the constitution of the cell in question, and may vary between cell types, and indeed between mesothelioma cells of different phenotypes. This study is the first to our knowledge to investigate whether such differentiation-dependent variation accounts for differences in sensitivity between cell phenotypes.

### Selenite induced apoptosis and sarcomatoid cells were more sensitive

**Figure 1:**
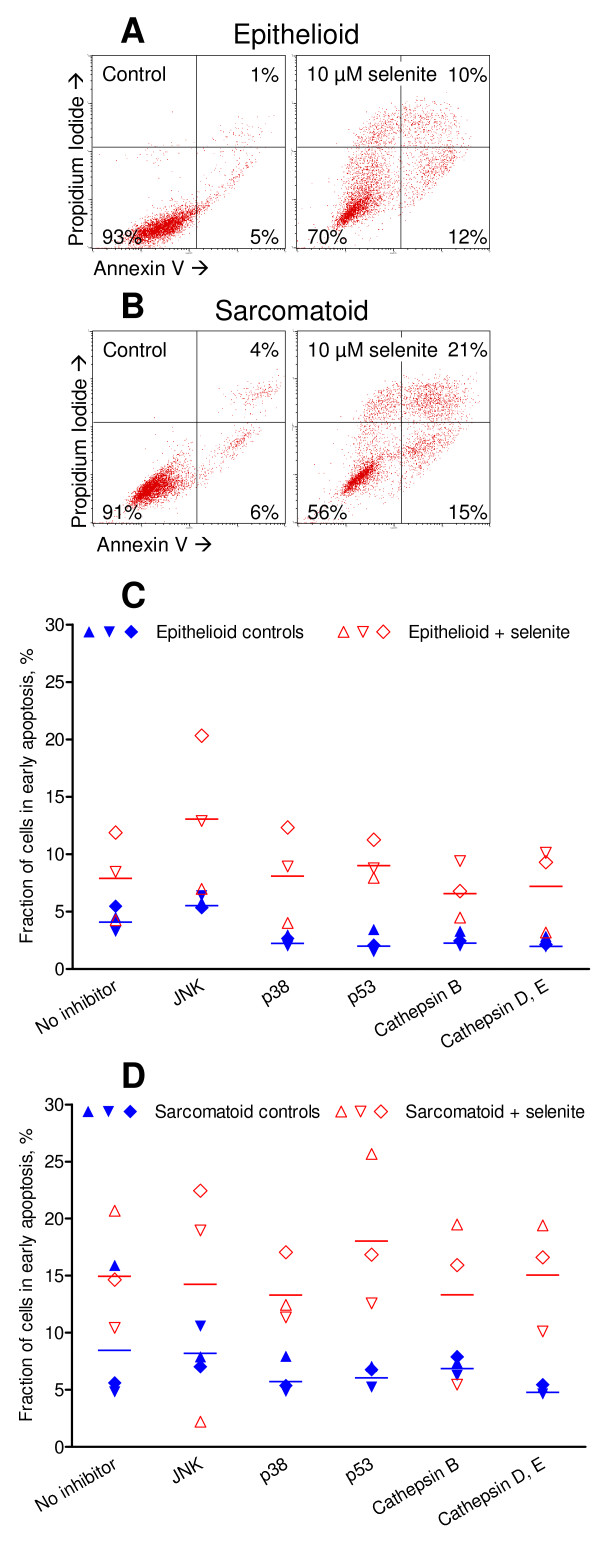
**Selenite-induced apoptosis as determined by the Annexin-PI assay and effects of inhibition of apoptosis signalling enzymes**. A and B: Representative Annexin-PI dot plots with typical distribution patterns and gating after 24 h treatment. Cells in the lower left quadrant are viable, those in the lower right quadrant are early apoptotic, and those in the upper quadrants are late apoptotic or necrotic. Early apoptosis in epithelioid (C) and sarcomatoid cells (D) before and after exposure to selenite for 24 h. Three independent experiments are shown. Two-way ANOVA with Dunnett's post test was performed to determine statistical significance between cells treated with selenite only and selenite with the respective inhibitors.

More than 90% of the untreated cells were viable, appearing in the lower left quadrant. Representative Annexin-PI plots are shown in figures 1A and 1B. Baseline early apoptosis (cells in the lower right quadrant), averaged over three experiments, was 4% in the epithelioid cells (Figure 1C) and 9% in the sarcomatoid cells (Figure 1D). Note that the figures 1A and 1B show only one representative experiment. 10 μM selenite decreased the viable fraction particularly in the sarcomatoid cells, as has also been reported previously [1]. This finding was confirmed by viability assays (data not shown). Selenite also increased the proportion of early apoptotic cells (Figures 1C and 1D). There were around 15% of cells in the upper quadrants in both cell types after selenite treatment, representing cells late in their apoptotic process or undergoing necrosis. A time-course experiment with measurements up to 48 h was performed to verify that 24 h was a suitable time-point for analysis (data not shown).

Loss of mitochondrial membrane potential (δΦ_m_) is associated with apoptosis. Following selenite treatment, a 3-fold loss of δΦ_m _was seen in both cell types (Table 2). These findings were confirmed by DiOC_6_(3) staining, and the specificity for mitochondria was verified using confocal microscopy (data not shown). The loss of δΦ_m _in both phenotypes after selenite treatment agrees well with earlier studies [15,19,36].

**Table 2:** Selenite-induced loss of mitochondrial membrane potential and effects of inhibition of apoptosis signalling enzymes

	***Epithelioid cells***			***Sarcomatoid cells***		
*Inhibitor*	*Loss of δΦ*_*m*_* after selenite treatment*^*a*^	*Statistical significance vs. no selenite*^*b*^	*Statistical significance vs. selenite only*^*c*^	*Loss of δΦ*_*m*_* after selenite treatment*^*a*^	*Statistical significance vs. no selenite*^*b*^	*Statistical significance vs. selenite only*^*c*^
Positive control	2.89 (± 0.68)			1.28 (± 0.18)		
Selenite	3.41 (± 0.57)	p < 0.01		3.30 (± 0.24)	p < 0.001	
JNK	0,94 (± 0.06)			1.05 (± 0.05)		
JNK + selenite	3,96 (± 0.58)	p < 0.001	ns	3.74 (± 0.25)	p < 0.001	ns
p38	0.99 (± 0.04)			0.88 (± 0.03)		
p38 + selenite	4.06 (± 0.63)	p < 0.001	ns	4.15 (± 0.52)	p < 0.001	ns
p53	0.74 (± 0.05)			0.92 (± 0.03)		
p53 + selenite	2.62 (± 0.57)	p < 0.05	ns	3.59 (± 0.52)	p < 0.001	ns
Cathepsin B	1.27 (± 0.12)			1.46 (± 0.10)		
Cathepsin B + selenite	5.68 (± 0.70)	p < 0.001	ns	6.27 (± 0.75)	p < 0.001	p < 0.01
Cathepsin D, E	0.93 (± 0.06)			0.90 (± 0.03)		
Cathepsin D, E + selenite	3.95 (± 0.77)	p < 0.001	ns	3.45 (± 0.37)	p < 0.001	ns

a: Fold change in JC-1 green fluorescence. Range shows the standard error of the mean (SEM).

b: One-way ANOVA analyses were performed with Bonferroni's multiple comparisons test.

c: One-way ANOVA analyses were performed with Dunnett's post test. ns = not significant.

To further delineate the role of signalling molecules among the MAP kinases and cathepsins, chemical inhibitors against these enzymes were used (Table 1). In the untreated epithelioid cells, the inhibitors decreased the baseline apoptotic fraction by 20–50% [see Additional file 1]. This demonstrates the efficacy of the inhibitors at the concentrations in which they were used. None of the enzyme inhibitors affected the proportion of viable cells during Annexin-PI apoptosis assays, although the WST-1 viability assays indicated a modest growth inhibitory effect of CA 074-Me and SB 203580 (data not shown).

Further controls to verify the efficacy of the chemical inhibitors were obtained by testing them on Jurkat cells over a 25 h time course following apoptosis induction with 0,2 μM staurosporine. The inhibitors of JNK, p53 and cathepsin D and E successfully decreased the apoptosis induction, whereas the cathepsin B inhibitor increased it [see Additional file 2].

### p38 inhibition reduced apoptosis frequency slightly in sarcomatoid cells

In the sarcomatoid cells, the p38 inhibitor SB203580 caused a small decrease in the apoptotic response to selenite (Figure 1D). In the epithelioid cells, p38 inhibition had no effect on the ability of selenite to induce apoptosis. However, selenite caused an even more marked drop of the δΦ_m _after p38 inhibition in both cell types (Table 2). This indicates that p38 was involved in apoptotic signalling particularly in the more sensitive sarcomatoid cells. The effect of inhibition was small however, and it cannot be regarded a key pathway. Activation of p38 after selenite exposure has previously been shown in cervix [18], leukemia [42] and prostate cancer cells [5].

### Inhibition of JNK increased the apoptotic response of epithelioid cells

Inhibition of JNK increased the proportion of selenite-induced early apoptotic cells by more than two thirds in the epithelioid cells (Figure 1C). In the sarcomatoid cells the effect was comparable to that without the inhibitor (Figure 1D). Scant effect on the loss of δΦ_m _was observed (Table 2). JNK apparently played no role in apoptosis signalling in the sarcomatoid cells. In the epithelioid cells, JNK even had a small antiapoptotic effect. The lack of proapoptotic activity is concordant with earlier findings in cervix cancer cells [18] but different from findings in prostate cancer cells [5].

### Selenite caused nuclear accumulation but inactivation of p53

**Figure 2:**
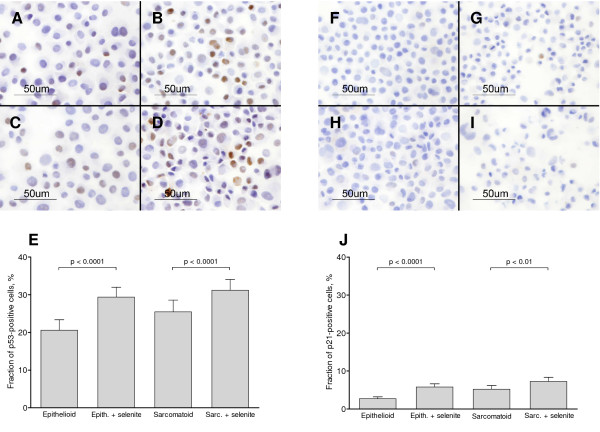
**Nuclear translocation of p53 and p21**. A-E: Immunocytochemical analysis of p53 performed on cytospin samples. A: Epithelioid cells without selenite. B: Epithelioid cells treated with 10 μM selenite for 24 h. C: Sarcomatoid cells without selenite. D: Sarcomatoid cells treated with 10 μM selenite for 24 h. E: Fraction of cells with p53-positive nuclei after 24 h, as assessed by two independent observers. Bars show the 95% confidence interval. χ^2^-tests were employed. F-J: Immunocytochemical analysis of p21 performed on cytospin samples, as an additional readout for p53 activity. F: Epithelioid cells without selenite. G: Epithelioid cells treated with 10 μM selenite for 24 h. H: Sarcomatoid cells without selenite. I: Sarcomatoid cells treated with 10 μM selenite for 24 h. J: Fraction of cells with p21-positive nuclei after 24 h, as assessed by three independent observers. Bars show the 95% confidence interval. χ^2^-tests were employed. Three independent experiments were performed.

**Figure 3:**
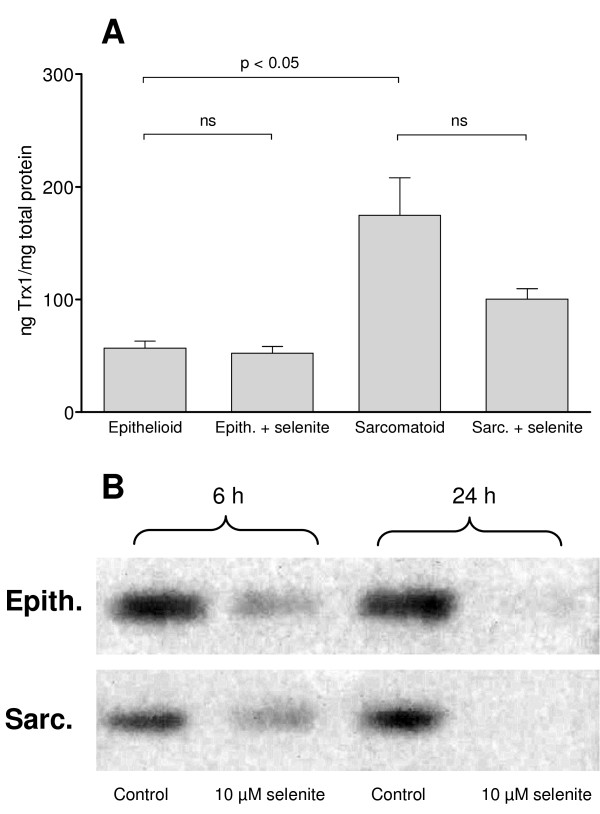
**Thioredoxin levels and p53 activity**. A: Amount of thioredoxin relative to total protein amount after 24 h. B: EMSA for p53 demonstrating less DNA-binding activity of p53 after selenite treatment in both cell types, but more markedly so in the epithelioid cells. Bars indicate the standard error of the mean. Student's t-test was performed. ns = not significant. Three independent experiments were performed.

Immunocytochemistry revealed that both epithelioid and sarcomatoid cells responded to selenite with a time-dependent increase of nuclear p53 immunoreactivity. After 24 h, the proportion of positive cells was increased approximately 1.5-fold (Figure 2A–E), and after 48 h, approximately 2-fold (not shown). EMSA analysis showed, however, that p53 exhibited less binding to DNA after selenite treatment (Figure 3B). Thus, although selenite caused nuclear accumulation of p53, it also decreased the DNA-binding activity. This result was surprising, as p53 has been implicated as a mediator of selenite-induced apoptosis signalling in other cell systems [5,17,18,43,44].

The expression of p21 (also known as Cip1 and WAF1) in response to genotoxic stress is tightly regulated by p53 (reviewed in [45]), and we therefore measured it as an additional indicator of p53 activity. The fraction of p21-positive cells was approximately doubled by selenite treatment (Figure 2F–J). Although these changes are statistically significant, the positive fraction was very small even after selenite treatment. As a positive control, epithelioid cells were treated with 2 μM doxorubicin and showed a 22% positive fraction (not shown).

Cells of either phenotype treated with the p53 inhibitor Pifithrin did not show a decreased apoptosis frequency as judged by Annexin-PI (Figure 1), nor a smaller loss of δΦ_m _(Table 2). This is particularly interesting since p53 inhibition decreased the baseline apoptosis in untreated cells (Figure 1, Additional file 1). Consequently, p53 was active in the control cells but was inactivated by selenite. Apoptosis was still induced by selenite, implicating p53-independent pathways in this process.

To find the mechanism of inhibition, we considered the complex regulation of p53 activity. The central DNA-binding core domain of p53 contains one zinc atom. Zinc chelators have been shown to cause accumulation of wild-type p53 in a structurally aberrant form with inhibited DNA-binding activity [46]. Selenium is a known chelator of zinc and when applied *in vivo *as selenite or its reduced form selenide, it forms nanocrystals of zinc-selenium with free or loosely bound zinc [47]. Another possibility is that selenite as an oxidizing agent may act directly upon regulatory cysteines on the p53 molecule, leading to an accumulation of oxidized p53 incapable of DNA-binding [48]. Also, secondary mediated redox regulation needs to be considered. The multifunctional protein Redox Effector Factor 1 (Ref-1) is involved in the redox regulation of stress inducible transcription factors such as Activating Protein-1, Nuclear Factor-κB, Hypoxia Inducible Factor-1 and p53, and may play an important role in this system. Ref-1 depends on thioredoxin (Trx) to maintain its active reduced state [49-51]. In a yeast experimental system, it has been shown that deletion of thioredoxin reductase (TrxR) downregulates p53 activity by keeping it in its oxidized form [52,53]. Trx overexpression on the other hand has been shown to increase p53 transactivation of reporter genes in human cell lines [49].

Protein levels of Trx were reduced by selenite treatment in sarcomatoid cells, from 175 ng/mg to 100 ng/mg. The epithelioid cells had a baseline expression of 57 ng/mg, decreasing slightly to 52 ng/mg after selenite treatment (Figure 3). It has been demonstrated previously that levels of TrxR decrease significantly in both cell lines with selenite treatment [1]. Consequently, redox inactivation of p53 is a plausible explanation for the lack of activity that was seen despite nuclear accumulation following selenite exposure.

### Selenite induced Bax up-regulation and Bcl-XL down-regulation

**Figure 4:**
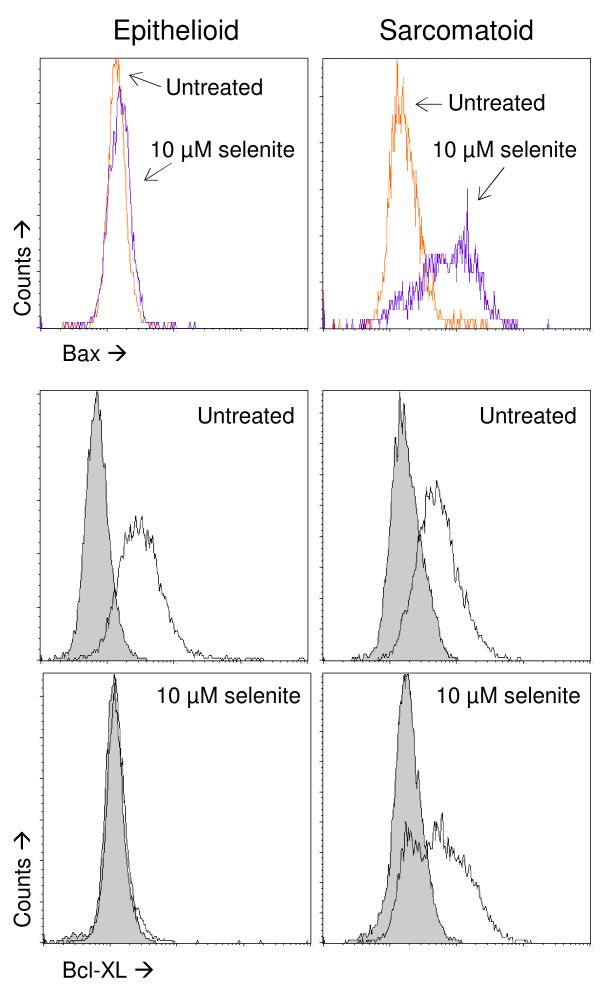
**Expression of Bax and Bcl-XL**. Top two panels: flow cytometric analyses of Bax expression. Sarcomatoid but not epithelioid cells responded to selenite treatment with a marked upregulation. Bottom four panels: flow cytometric analyses of Bcl-XL expression. Epithelioid cells lost Bcl-XL expression completely after selenite treatment, whereas sarcomatoid cells showed a partial loss. Gray histograms show the negative controls for the immunostaining. Three independent experiments were performed.

The immunoreactivity for the proapoptotic mediator Bax increased significantly in the sarcomatoid cells but not in the epithelioid cells following selenite treatment (Figure 4). This clear phenotypic difference may partially explain why sarcomatoid cells are more sensitive to selenite. Morphological controls verified that staining was localised to cytoplasmic granules consistent with mitochondria (not shown). Although activation of Bax in response to selenite has been shown in other systems [9,17,18,44,54], this is the first report of differential expression coupled to sensitivity.

In mesothelioma, the antiapoptotic Bcl-2 family member Bcl-XL is frequently overexpressed [55], and this has been shown to be an important mechanism by which mesothelioma cells gain apoptosis resistance [56]. In the epithelioid cells, the Bcl-XL expression decreased markedly after selenite treatment, whereas only a subpopulation of the sarcomatoid cells showed lower expression after treatment (Figure 4).

### Selenite caused caspase activation particularly in the epithelioid cells

**Figure 5:**
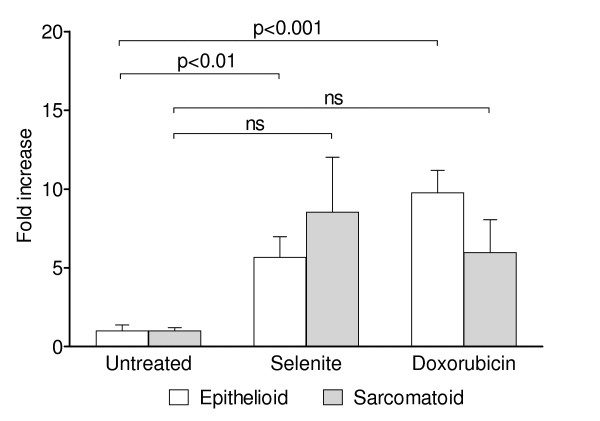
**Caspase activation as determined by cytokeratin 18 cleavage**. M-30 Apoptosense assay showing the amounts of caspase-cleaved cytokeratin 18 fragments detected. Bars indicate the standard error of the mean. For statistical analyses, two-way ANOVA with Dunnett's post test was used. Data represent the means of three independent experiments.

Both epithelioid and sarcomatoid cells showed a 6-fold increase in caspase-mediated cleavage of cytokeratin 18 after selenite treatment (Figure 5), indicating activation of caspases 3, 6, 7, and 9. Doxorubicin, as a positive control, caused a 10-fold increase in the epithelioid cells and a 6-fold increase in the sarcomatoid cells.

**Figure 6:**
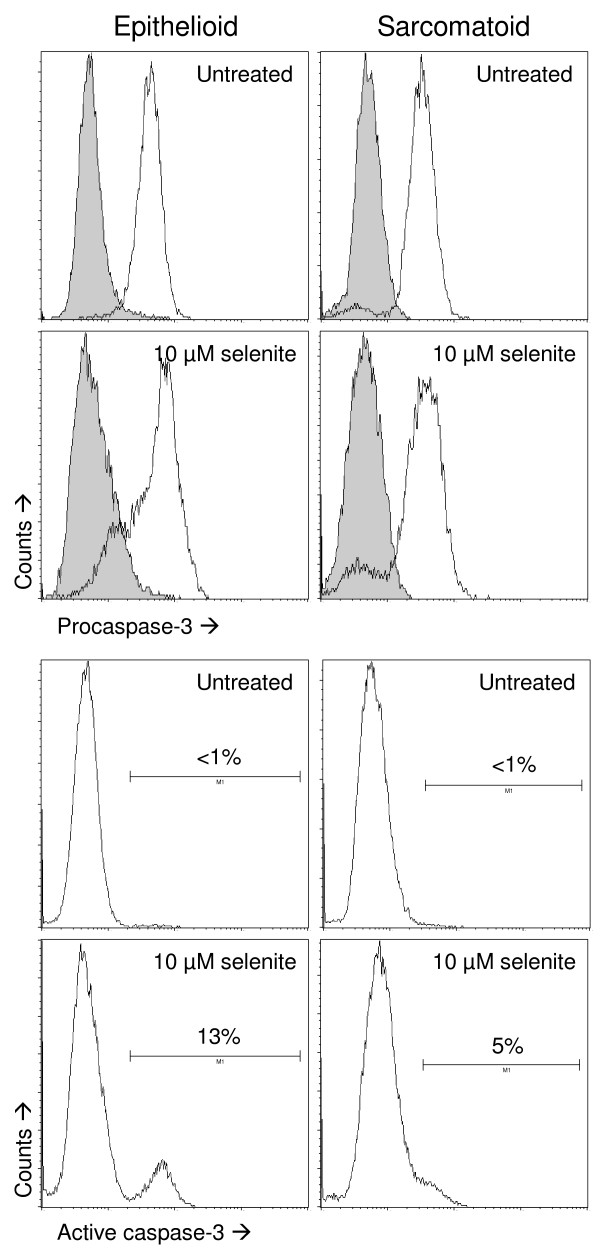
**Caspase-3 activation as determined by flow cytometry**. Top four panels: flow cytometric analyses of procaspase-3. Sarcomatoid and epithelioid cells showed a similar baseline expression. In both cell types, a subpopulation lost expression after selenite treatment. Gray histograms show the negative controls for the immunostaining. Bottom four panels: flow cytometric analyses of caspase-3 activation. Selenite treatment caused the appearance of a distinctly positive subpopulation in the epithelioid cells, whereas the sarcomatoid cells showed a small positive subpopulation that was not distinctly separated from the main peak. Three independent experiments were performed. All eight panels are derived from the same experiment.

Flow cytometric analysis for procaspase-3 showed that both cell types have a similar baseline expression. After selenite treatment, subpopulations of both phenotypes lose procaspase-3. In the epithelioid cells, this corresponds to the appearance of a distinct subpopulation (13%) that is positive for activated caspase-3. In the sarcomatoid cells, there is also a small fraction (5%) of cells that stain more intensely for activated caspase-3, but it is not distinctly separated from the main peak (Figure 6). Caspase-3 activation was confirmed in doxorubicin treated-controls: 49% of epithelioid cells were positive, and 20% of sarcomatoid cells (not shown).

Divergent data have been published regarding the role of caspases in selenite-induced apoptosis. Several studies have shown that selenite causes a caspase-independent apoptotic cell death [6,18,40], whereas others have shown caspase-dependence [9,17,36,57]. We report that caspase-3 was activated in a sub-population of epithelioid cells, but little reactivity was seen in sarcomatoid cells. The limited caspase activation in sarcomatoid cells was surprising. A possible explanation could be an upregulation of Inhibitor of Apoptosis (IAP) family members such as survivin and XIAP. Earlier studies have found that overexpression of IAP family members is common in mesothelioma cells [58-61].

### Inhibition of cathepsin B but not of cathepsins D and E caused increased loss of δΦ_m_

Cathepsins are a group of proteases that are physiologically present in lysosomes, and may be released upon stimuli such as oxidative stress [62]. Cells that were pretreated with cathepsin B inhibitor CA-074 Me showed slightly less apoptosis after selenite exposure (Figure 1). In the sarcomatoid cells, this was reflected in correspondingly increased viability. In the epithelioid cells, the viable proportion decreased slightly instead. Interestingly, when selenite was combined with the cathepsin B inhibitor, the loss of δΦ_m _was greater than with any other inhibitor (Table 2). Cathepsin D and E inhibitor Pepstatin A did not affect the induction of apoptosis by selenite, nor did it alter the loss of δΦ_m_.

### Signs of autophagy were not detected

Autophagy is a form of programmed cell death in which cells do not exhibit apoptotic characteristics. Kim et al have shown that selenite induces autophagy in glioma cells [38]. We wanted to investigate whether some of the cell death that we observe could be due to autophagy. Cells were stained with monodansyl cadaverine and analysed with confocal microscopy for the appearance of granules that might represent autophagic vesicles. No changes in the staining pattern could however be detected between untreated and selenite-treated cells of either phenotype [see Additional file 3].

## Concluding remarks

Our results clearly demonstrate that selenite causes a complex pattern of cell death in malignant mesothelioma cells. Selenite causes both apoptosis and necrosis, but cells exhibiting apoptotic characteristics such as Annexin V externalisation do not necessarily display other classical apoptosis-related changes such as caspase-activation [6,18,40]. It appears purposeful to consider selenite-induced cell death to lie on a spectrum between apoptosis and necrosis, where the exact mode of cell death differs depending on phenotype characteristics.

Our results indicate that mesothelioma cells activate p38 and JNK in response to selenite, and that they accumulate p53 in the nucleus, but in a form bereft of DNA-binding activity. We hypothesise that this interesting phenomenon is due to a shift in redox balance towards a prooxidative state with increased levels of reactive oxygen species (ROS) and a loss of thioredoxin system activity.

Sarcomatoid mesothelioma cells, although ordinarily chemoresistant, are more sensitive to selenite than epithelioid cells [1]. The differential activation of apoptosis-signaling proteins on the level of the mitochondrion may partially explain the observed differences in sensitivity.

A better understanding of the proapoptotic mechanisms of selenite as well as of phenotype-dependent response patterns in mesothelioma cells will aid the development of cancer therapies with greater efficacy and which may be better suited to the diverse biology of individual tumors. Malignant mesothelioma is a heterogeneous entity, and further studies on differentiation-related sensitivity to selenite and other cytotoxic drugs are under way in our laboratory using a panel of cell lines of varying epithelioid-sarcomatoid differentiation.

## Competing interests

The authors declare that they have no competing interests.

## Authors' contributions

GN participated in the study design, conducted most of the experiments with cell viability assays, flow cytometry, immunocytochemistry, and confocal microscopy, performed the data analysis, participated in the interpretation of results, and drafted the manuscript. EO performed the EMSA and Trx analyses. ASz and FM participated in the cell viability and flow cytometric experiments. ASt and BK participated in the immunocytochemical experiments. AKR and APF participated in the interpretation of results and in performing the Trx analyses. MB participated in the study design and in the interpretation of results. KD was responsible for the overall study design, participated in the flow cytometric and immunocytochemical experiments, in the interpretation of results, and helped draft the manuscript. All authors read and approved the final manuscript.

## Supplementary Material

Additional file 1 **Internal verification of the efficacy of apoptosis signalling enzyme inhibitors**. An internal verification of the efficacy of the inhibitors was established by their ability to reduce apoptosis in the control cells. Two-way ANOVA with Dunnett's post test was used to compare the apoptosis frequency with the respective inhibitors to that in the control cells without any inhibitor. Asterisks denote p < 0.05. Data represent the same three independent experiments illustrated in figure 1. Bars indicate the standard error of the mean. Click here for file

Additional file 2 **External verification of the efficacy of apoptosis signalling enzyme inhibitors**. A-E: Apoptosis kinetics of Jurkat cells treated with staurosporine and chemical inhibitors, to verify that the inhibitors were able to alter the apoptotic rate. Graphs show the proportion of early apoptotic cells as determined by flow cytometry. Numbers given on the graphs show the area under the respective curves. One experiment was performed. Click here for file

Additional file 3 **Monodansyl cadaverine staining for autophagy**. A-D: Confocal micrographs of cells stained with MDC. A: Epithelioid cells, untreated. B: Epithelioid cells, treated with 10 μM selenite for 24 h. C: Sarcomatoid cells, untreated. D: Sarcomatoid cells, treated with 10 μM selenite for 24 h. In all cases, staining is seen in the endoplasmic reticulum surrounding the nucleus, with no evidence of granular structures that might represent autophagic vesicles. Bars are 50 μm. Three independent experiments were performed. Click here for file
