# Supplementary figures and images for: Phenotype-dependent apoptosis signalling in mesothelioma cells after selenite exposure
Source: J Exp Clin Cancer Res. 2009 Jun 29;28(1):92. doi: 10.1186/1756-9966-28-92 (PMC2711967; doi:10.1186/1756-9966-28-92)

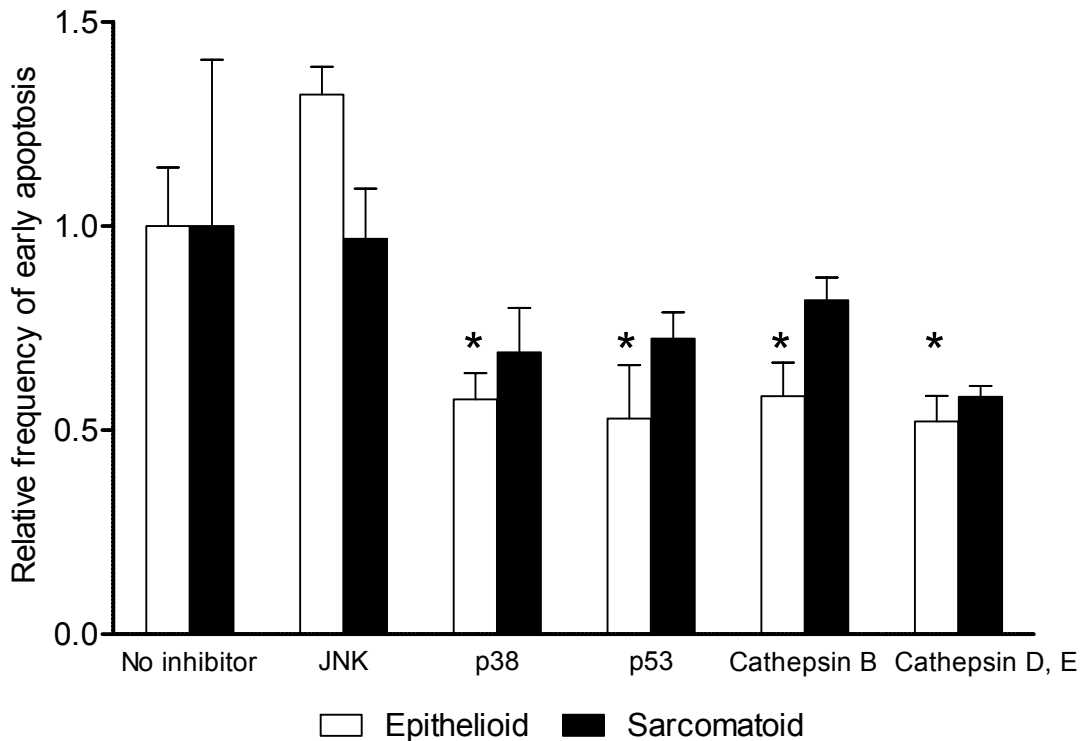

Supplement: Additional file 1 — Internal verification of the efficacy of apoptosis signalling enzyme inhibitors. An internal verification of the efficacy of the inhibitors was established by their ability to reduce apoptosis in the control cells. Two-way ANOVA with Dunnett's post test was used to compare the apoptosis frequency with the respective inhibitors to that in the control cells without any inhibitor. Asterisks denote p < 0.05. Data represent the same three independent experiments illustrated in figure 1. Bars indicate the standard error of the mean. [file 1756-9966-28-92-S1.pdf]

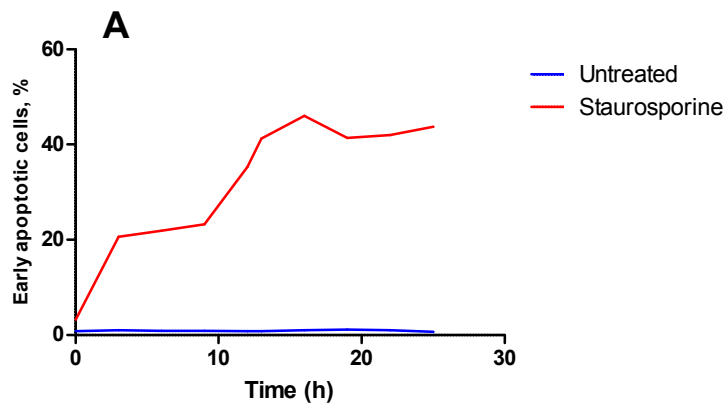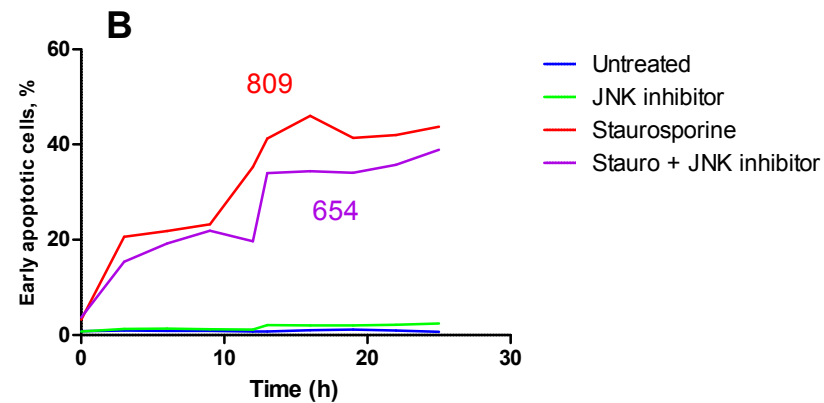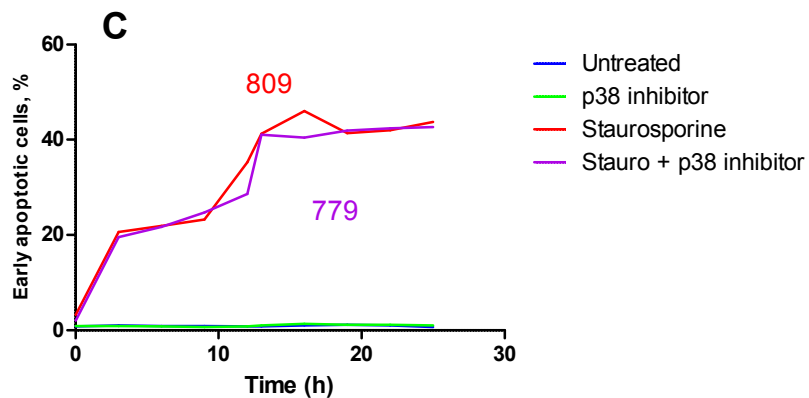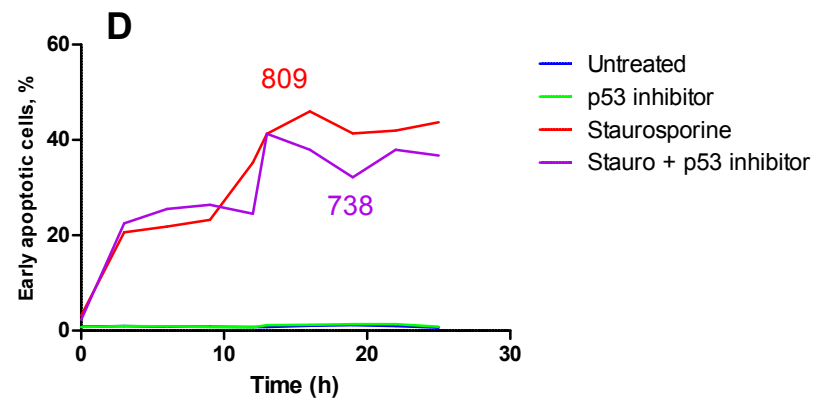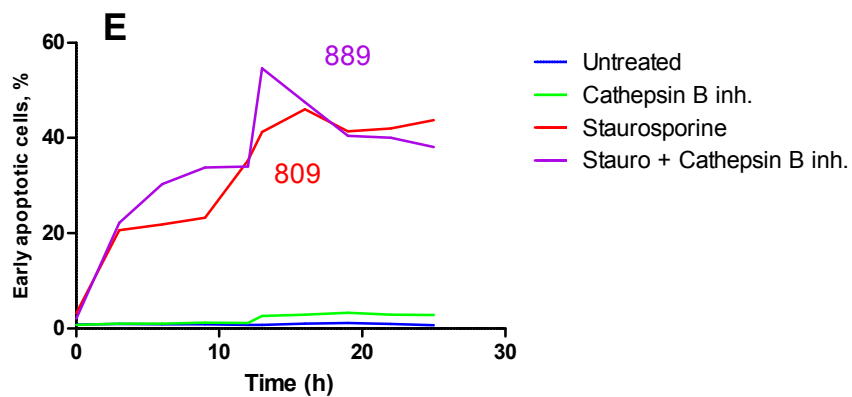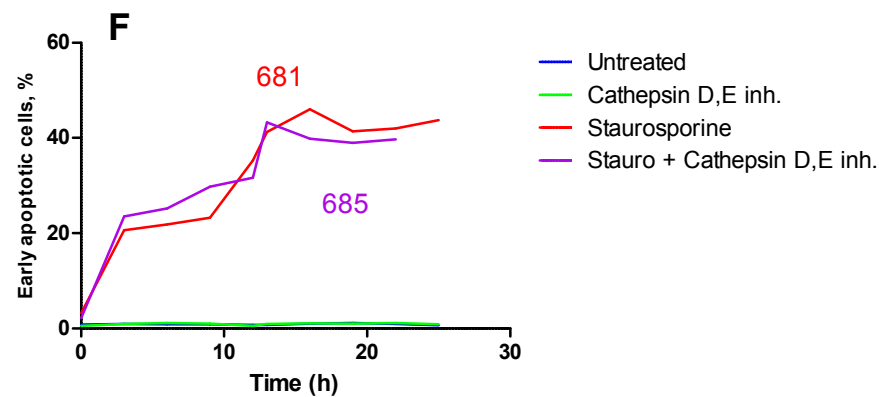

Supplement: Additional file 2 — External verification of the efficacy of apoptosis signalling enzyme inhibitors. A-E: Apoptosis kinetics of Jurkat cells treated with staurosporine and chemical inhibitors, to verify that the inhibitors were able to alter the apoptotic rate. Graphs show the proportion of early apoptotic cells as determined by flow cytometry. Numbers given on the graphs show the area under the respective curves. One experiment was performed. [file 1756-9966-28-92-S2.pdf]

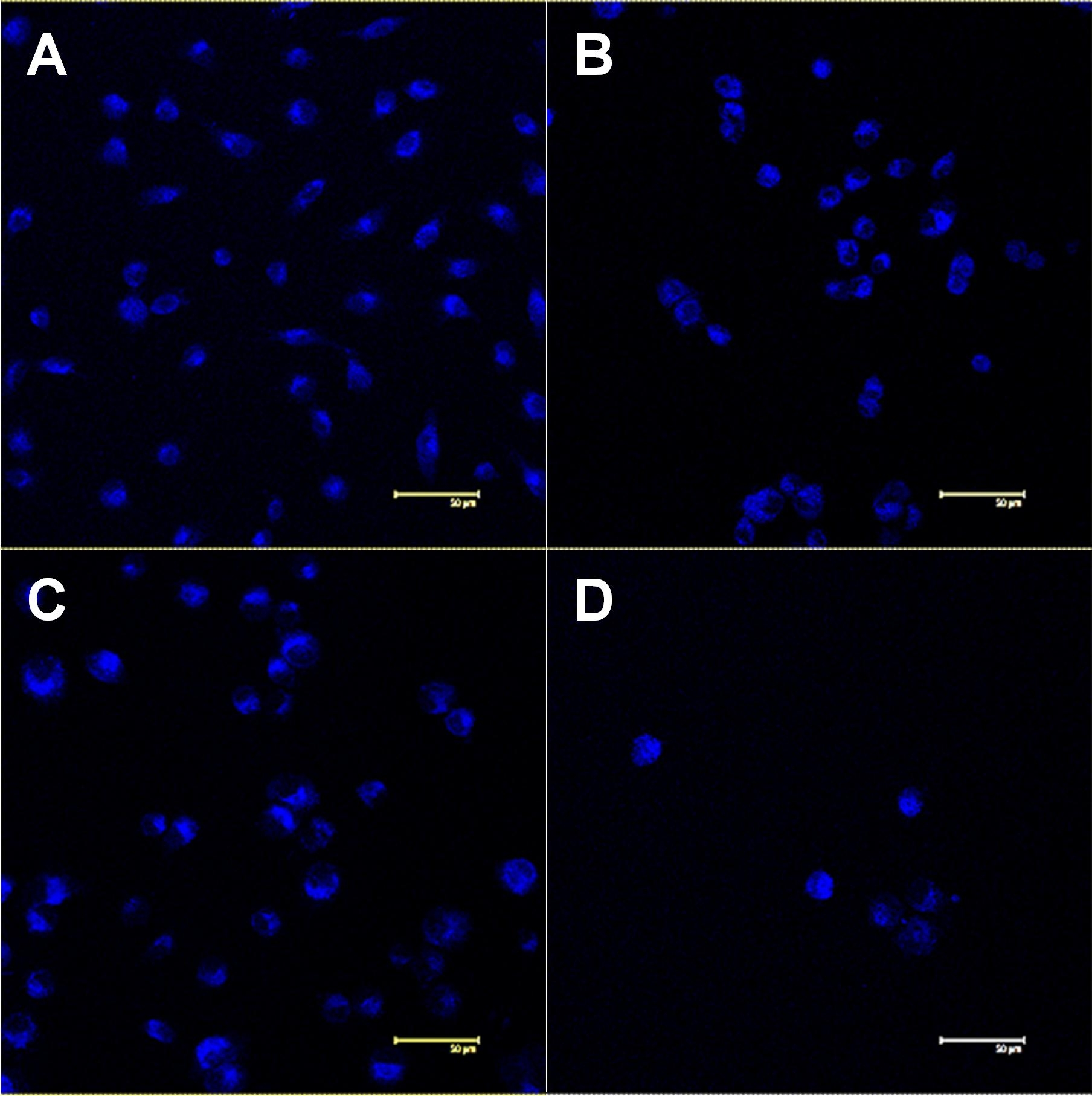

Supplement: Additional file 3 — Monodansyl cadaverine staining for autophagy. A-D: Confocal micrographs of cells stained with MDC. A: Epithelioid cells, untreated. B: Epithelioid cells, treated with 10 μM selenite for 24 h. C: Sarcomatoid cells, untreated. D: Sarcomatoid cells, treated with 10 μM selenite for 24 h. In all cases, staining is seen in the endoplasmic reticulum surrounding the nucleus, with no evidence of granular structures that might represent autophagic vesicles. Bars are 50 μm. Three independent experiments were performed. [file 1756-9966-28-92-S3.jpeg]
